# ADGRL4/ELTD1 Silencing in Endothelial Cells Induces ACLY and SLC25A1 and Alters the Cellular Metabolic Profile

**DOI:** 10.3390/metabo9120287

**Published:** 2019-11-25

**Authors:** David M. Favara, Christos E. Zois, Syed Haider, Elisabete Pires, Helen Sheldon, James McCullagh, Alison H. Banham, Adrian L. Harris

**Affiliations:** 1 Balliol College, University of Oxford, Oxford OX1 3BJ, UK; 2 Department of Oncology, University of Oxford, Oxford OX3 7DQ, UK; christos.zois@oncology.ox.ac.uk (C.E.Z.); Syed.Haider@icr.ac.uk (S.H.); helen.sheldon@oncology.ox.ac.uk (H.S.); 3 Breast Cancer Now Toby Robins Research Centre, The Institute of Cancer Research, London SW7 3RP, UK; 4 Chemistry Research Laboratory, Department of Chemistry, University of Oxford, Oxford OX1 3TA, UK; elisabete.pires@chem.ox.ac.uk (E.P.); james.mccullagh@chem.ox.ac.uk (J.M.); 5 Nuffield Division of Clinical Laboratory Science, Radcliffe Department of Medicine, University of Oxford, Oxford OX3 9DU, UK; alison.banham@ndcls.ox.ac.uk

**Keywords:** ADGRL4/ELTD1, metabolomics, adhesion GPCR

## Abstract

Adhesion G Protein-Coupled Receptor L4 (ADGRL4/ELTD1) is an endothelial cell adhesion G protein-coupled receptor (aGPCR) which regulates physiological and tumour angiogenesis, providing an attractive target for anti-cancer therapeutics. To date, ADGRL4/ELTD1′s full role and mechanism of function within endothelial biology remains unknown, as do its ligand(s). In this study, ADGRL4/ELTD1 silencing, using two independent small interfering RNAs (siRNAs), was performed in human umbilical vein endothelial cells (HUVECS) followed by transcriptional profiling, target gene validation, and metabolomics using liquid chromatography-mass spectrometry in order to better characterise ADGRL4/ELTD1′s role in endothelial cell biology. We show that ADGRL4/ELTD1 silencing induced expression of the cytoplasmic metabolic regulator ATP Citrate Lyase (ACLY) and the mitochondria-to-cytoplasm citrate transporter Solute Carrier Family 25 Member 1 (SLC25A1) but had no apparent effect on pathways downstream of ACLY (fatty acid and cholesterol synthesis or acetylation). Silencing induced KIT expression and affected the Notch signalling pathway, upregulating Delta Like Canonical Notch Ligand 4 (DLL4) and suppressing Jagged Canonical Notch Ligand 1 (*JAG1*) and Hes Family BHLH Transcription Factor 2 (*HES2*). The effect of ADGRL4/ELTD1 silencing on the cellular metabolic profile was modest but several metabolites were significantly affected. Cis-aconitic acid, uridine diphosphate (UDP)-glucoronate, fructose 2,6-diphosphate, uridine 5-diphosphate, and aspartic acid were all elevated as a result of silencing and phosphocreatine, N-acetylglutamic acid, taurine, deoxyadenosine triphosphate, and cytidine monophosphate were depleted. Metabolic pathway analysis implicated ADGRL4/ELTD1 in pyrimidine, amino acid, and sugar metabolism. In summary, this study shows that ADGRL4/ELTD1 impacts core components of endothelial metabolism and regulates genes involved in endothelial differentiation/homeostasis and Notch signalling.

## 1. Introduction

**Figure 1:**
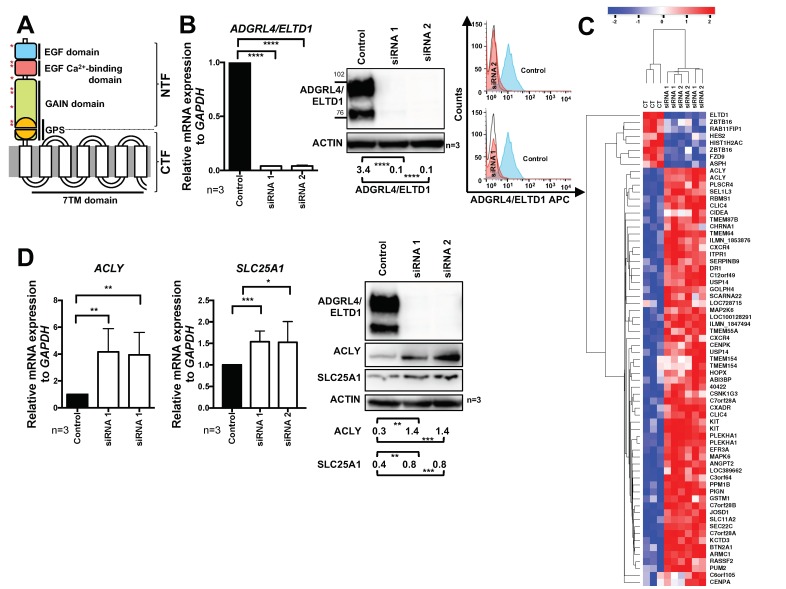
ADGRL4/ELTD1 silencing induces ACLY and SLC25A1 expression. (**A**) ADGRL4/ELTD1′s putative structure. Glycosylation sites are indicated by red asterisks. (**B**) Validation of ADGRL4/ELTD1 silencing in human umbilical vein endothelial cells (HUVECs): qPCR (mRNA), representative Western blot (protein; the double bands reflect glycosylation posttranslational modification [1]), and representative FACS (for cell-surface protein). (**C**) Heatmap of 68 significant differentially expressed genes following ADGRL4/ELTD1 silencing. Colouring represents the z-score for each gene (low = blue; high = red). (**D**) Validation of ACLY and SLC25A1 expression. qPCR and representative Western blot (* *p* ≤ 0.05, ** *p* ≤ 0.01, *** *p* ≤ 0.001, **** *p* ≤ 0.0001). Legend: GPS, G protein-coupled receptor (GPCR) proteolysis site; EGF, epidermal growth factor.

ADGRL4/ELTD1 is an orphan adhesion G protein-coupled receptor (GPCR) of clinical and therapeutic interest due to its regulation of physiological and tumour angiogenesis [1]. ADGRL4/ELTD1 belongs to the 33-member adhesion GPCR (aGPCR) family, one of the five GPCR families making up the GPCR superfamily. The aGPCR family contains large extracellular domains with adhesion motifs (a feature not found in other GPCR families) [2]. It is also the most poorly studied and understood of the five GPCR families [3,4]. *ADGRL4/ELTD1* arose approximately 435 million years ago in the first vertebrates and forms part of the early angiogenesis gene repertoire [5]. No X-ray crystal structures have been obtained but like all adhesion GPCRs, ADGRL4/ELTD1′s predicted structure comprises an N-terminal fragment (NTF) and a C-terminal fragment (CTF) which are centred around the GPCR proteolysis site (GPS), a site which is cleaved by autoproteolysis and thereafter non-covalently re-joined during protein assembly [6] (Figure 1A). ADGRL4/ELTD1′s adhesion domains comprise an epidermal growth factor (EGF) repeat followed by an EGF-Ca2+ binding repeat, the latter being highly conserved across ADGRL4/ELTD1 orthologues, suggesting it has functional importance [5].

ADGRL4/ELTD1 is expressed within endothelial cells and vascular smooth muscle cells and is upregulated within tumour-associated endothelial cells across a range of tumour types (head and neck, renal, colorectal, and ovarian cancer) [1]. It is differentially regulated by two key angiogenic ligands (Vascular Endothelial Growth Factor (VEGF) (upregulation) and DLL4 (downregulation)) and has an important role in regulating sprouting angiogenesis, with silencing disrupting vessel formation both in vitro and in vivo [1]. In zebrafish embryos, ADGRL4/ELTD1 silencing causes lethality [1]; however, this does not happen in mice [7], suggesting the presence of added genetic redundancy. In mice, systemic ADGRL4/ELTD1 silencing causes a reduction in size of colorectal and ovarian tumour xenografts (without toxicity) and improves survival [1]. In human patients with colorectal and ovarian cancer who go on to receive systemic anti-cancer therapy, high tumour-associated endothelial ADGRL4/ELTD1 expression correlates with improved overall survival in a range of tumour types (head and neck squamous carcinoma, renal, colorectal, ovarian, and hepatocellular cancers) [1,8]. In these tumours, it has been shown to have importance in blood vessel development, hence the correlation with higher expression (higher tumour micro-vessel density) and survival when patients are given anti-cancer therapy. ADGRL4/ELTD1 is not expressed by the majority of cancer cell lines [9]. However, it is expressed by glioblastomas where it possibly functions in a different way and is important for tumour survival, and is an emerging therapeutic target in this tumour type [10,11,12].

Taken together, these characteristics make ADGRL4/ELTD1 an attractive oncology clinical target. To further investigate its role in endothelial biology, we silenced ADGRL4/ELTD1 in human umbilical vein endothelial cells (HUVECS), examined the changes in gene expression, and, as enzymes and transporters involved in metabolism were induced, conducted a metabolic analysis.

## 2. Results

### 2.1. ADGRL4/ELTD1 Silencing Induces ACLY and SLC25A1 Expression and Affects Expression of KIT and Notch Pathway Genes

Primary HUVECs from three unique donor pools were silenced for ADGRL4/ELTD1 expression over 48 h using two different small interfering RNAs (siRNAS) (siRNA 1 and 2). Proof of effective silencing was determined at both the transcript and protein level (Figure 1B and Figure S1A). Global transcriptional profiling was then performed on biological replicates of these ADGRL4/ELTD1 silenced cells. This showed 68 genes with a greater than two-fold up/down-regulation common to both siRNAs (Figure 1C and Figure S1B). The gene whose expression was most highly induced by ADGRL4/ELTD1 silencing was *ACLY*, a core metabolic enzyme which converts cytoplasmic citrate into acetyl-coenzyme A (acetyl-CoA) (Figure 1D). Silencing also induced S*LC25A1,* a citrate transporter which transports citrate from the mitochondria into the cytoplasm where it acts as a substrate for ACLY (Figure 1D). Both *ACLY* and *SLC25A1* were upregulated at the mRNA and protein level (Figure 1D). The haematopoietic stem cell regulator KIT was also significantly upregulated at both at the mRNA and protein levels (Figure S1C). Furthermore, ADGRL4/ELTD1 silencing affected expression of the Notch pathway target genes, which are central to endothelial cell angiogenesis, upregulating DLL4 (Figure S1D), while suppressing *JAG1 (*Figure S1E) and *HES2* (Figure S1F). Of the above findings, only ADGRL4/ELTD1′s relationship with DLL was previously known [1]. ADGRL4/ELTD1 stable overexpression in HUVECs caused no significant change in *ACLY* or *SLC25A1* mRNA expression (Figure S1G).

### 2.2. ADGRL4/ELTD1 Silencing Does Not Affect Acetylation Reactions or Lipid Droplet Formation

**Figure 2:**
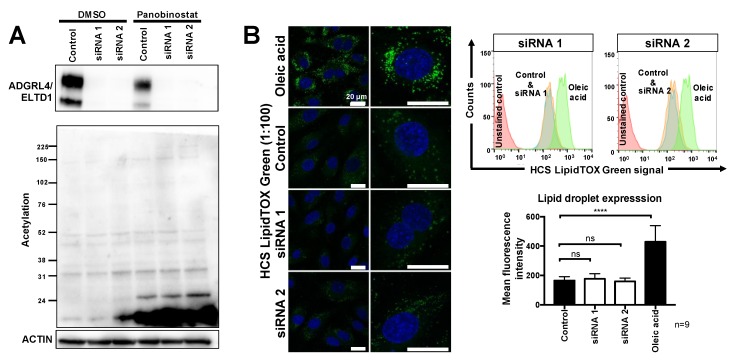
ADGRL4/ELTD1 silencing does not affect acetylation or lipid droplet formation. (**A**) Representative Western blot showing the effect of silencing on histone acetylation. Panoboninostat (25 nM) was used as a positive control for global acetylation. (**B**) Representative confocal microscopy showing effect of silencing on lipid droplets. Oleic acid (150 μM) was used a positive control. Representative FACS histograms and FACS quantification. (* *p* ≤ 0.05, ** *p* ≤ 0.01, *** *p* ≤ 0.001, **** *p* ≤ 0.0001).

Due to the upregulation of both SLC25A1 and ACLY, we investigated whether ADGRL4/ELTD1 silencing had an effect on the pathways downstream of ACLY, namely, (i) acetylation reactions, (ii) fatty acid synthesis, and (iii) cholesterol synthesis. Acetylation was investigated by screening for changes in global acetylation following ADGRL4/ELTD1 silencing (panobinostat, a pan-HDAC inhibitor, was used as an acetylation positive control). This showed that silencing had no effect on acetylation reactions and that panobinostat partially suppressed ADGRL4/ELTD1 expression, the first known non-siRNA negative regulator of ADGRL4/ELTD1 protein expression (Figure 2A). Lipid droplet formation, an outcome of both fatty acid and cholesterol synthesis, was investigated next (oleic acid was used as a positive control). This showed no changes in global lipid droplet accumulation in ADGRL4/ELTD1-silenced HUVECS using both confocal microscopy and FACS (Figure 2B). Furthermore, qPCR investigation of a panel of core fatty acid synthesis genes (Figure S2A) failed to show any significant change in gene expression caused by both *ADGRL4/ELTD1*-targeting siRNAs (Figure S2B).

### 2.3. ADGRL4/ELTD1 Silencing Affects Various Metabolic Pathways

**Figure 3:**
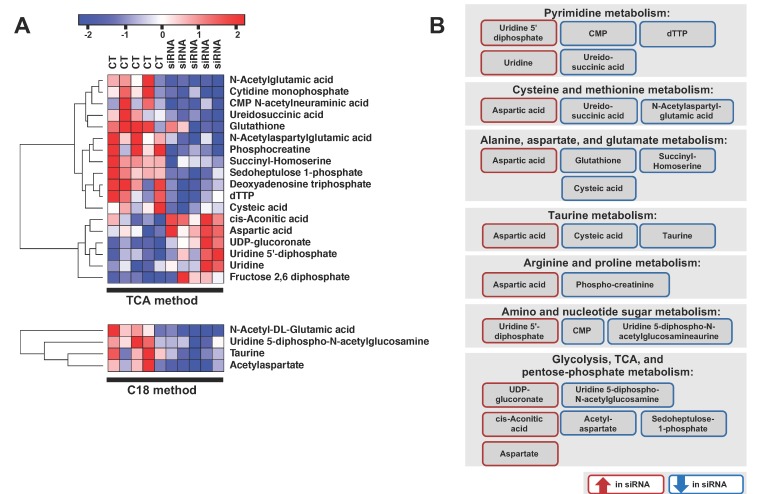
Metabolite changes following ADGRL4/ELTD1 silencing. (**A**) Metabolite heatmap using metabolites identified by liquid chromatography-mass spectrometry (LC-MS) (*p* ≤ 0.05 and fold change > 1.2). (**B**) Summary of enrichment analyses (red: upregulated metabolites, blue: downregulated metabolites). (Abbreviations: CMP=cytidine monophosphate; dTTP= deoxythymidine triphosphate; UDP= uridine diphosphate)

Next, we used metabolomics to investigate whether ADGRL4/ELTD1 had any effect on global HUVEC metabolism. This was performed by analysing ADGRL4/ELTD1-silenced HUVECS with liquid chromatography-mass spectrometry (LC-MS). Twenty-one metabolites were significantly altered (*p* < 0.05 with fold change (FC) > 1.2) (Figure 3A). The top five elevated metabolites were cis-aconitic acid (*p* = 0.01; FC = 1.67), UDP-glucoronate (*p* = 0.001; FC = 1.58), fructose 2,6 diphosphate (*p* = 0.01; FC 1.4), uridine 5-diphosphate (*p* = 0.03; FC = 1.3), and aspartic acid (*p* = 0.01; FC = 1.3). The top five depleted metabolites were phosphocreatine (*p* = 0.008; FC = −3.7), N-acetylglutamic acid (*p* = 0.0009; FC = −2.7), deoxyadenosine triphosphate (*p* = 0.01; FC = −2.1), taurine (*p* = 0.02; FC = −1.96), and cytidine monophosphate (*p* = 0.0005; FC = −1.8) (Figure 3A). N-acetylglutamic acid was the only significantly altered metabolite shared between LC-MS methods.). Significant metabolite changes (21 metabolites) are shown in Figure S3. All metabolomics output is available in the supplementary data file.

**Figure 4:**
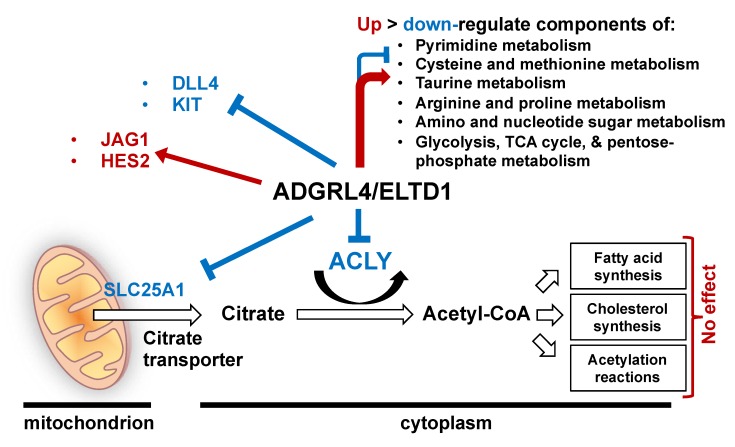
Graphical summary of ADGRL4/ELTD1′s effect on gene expression and metabolites.

Pathway analysis showed enrichment within pyrimidine metabolism (5/60; *p* < 0.0001), alanine aspartate and glutamine metabolism (4/56; *p* = 0.0008), cysteine and methionine metabolism (3/24; *p* = 0.009), taurine metabolism (2/20; *p* = 0.01), arginine and proline metabolism (3/77; *p* = 0.02), and amino and nucleotide sugar metabolism (3/88; *p* = 0.03) (Figure 3B). Non-significant enrichment was seen in glycolysis, the citric acid cycle, and the pentose-phosphate pathway (Figure 3B). As the number of samples analysed was low, pathway analysis was also performed on all LC-MS metabolites with a FC > 1.2, irrespective of *p* value. This showed enrichment in similar pathways (Table S1). Figure 4 depicts a summary of ADGRL4/ELTD1′s effect on gene expression.

## 3. Discussion

Our results reveal ADGRL4/ELTD1 as a regulator of endothelial metabolism and KIT expression and identify new relationships with the Notch pathway. The finding that ADGRL4/ELTD1 down-regulates ACLY and SLC25A1 expression is interesting as both encode critical enzymes in cellular metabolism [13,14]. Interestingly, ADGRL4/ELTD1 overexpression in HUVECs did not downregulate *ACLY* or *SLC25A1*, potentially because they are already maximally suppressed by endogenous levels of ADGRL4/ELTD1. Although ADGRL4/ELTD1 did not affect fatty acid or cholesterol synthesis or acetylation reactions, metabolomics showed that ADGRL4/ELTD1 has an effect on metabolites in multiple metabolic pathways, the most significant being pyrimidine metabolism. Through its conversion of citrate to acetyl-coA, cytoplasmic ACLY links glucose metabolism with both fatty acid and cholesterol synthesis pathways [14,15] whilst SLC25A1 traffics citrate from the citric acid cycle out of the mitochondria into the cytoplasm [16,17]. This citrate provides the acetyl units for lipogenesis and is catalysed by ACLY, producing acetyl-CoA which feeds fatty acid synthesis or cholesterol synthesis, or regulates glycolytic histone modification [18,19]. Endothelial cells depend on glycolysis for ATP generation [20] and use beta oxidation to generate carbon for endothelial DNA synthesis rather than using fatty acid oxidation as an alternative source of energy [21]. VEGFA, the initiating ligand for endothelial cell sprouting, increases endothelial glycolysis [20] and induces ADGRL4/ELTD1 expression [1]. T rans-differentiation of fibroblasts into induced endothelial cells requires a glycolytic switch involving increased expression of SLC25A1 and ACLY as well as the intracellular accumulation of citrate, suggesting that both are important in the development and maintenance of the endothelial cell metabolic phenotype [22] . Furthermore, complete ablation of ACLY induces the endothelial to mesenchymal transition (EMT), while increasing acetyl-coA suppresses EMT [23]. These findings suggest that ADGRL4/ELTD1’s regulation of ACLY and SLC25A1 expression may help maintain endothelial homeostasis and metabolism. It remains unclear why ADGRL4/ELTD1 silencing upregulates ACLY, but downstream acetylation and/or lipid droplet formation are unaffected. This is most likely due to tight regulation of glycolysis in endothelial cells due to the integral role of glycolysis in endothelial cell function [21,24].

ADGRL4/ELTD1 silencing induced the accumulation of cis-aconitate (a citric acid cycle intermediary), UDP-glucoronate (a sugar metabolism intermediary), fructose 2,6 diphosphate (a potent stimulator of glycolysis [25]), uridine 5-diphosphate (a glycogen metabolism intermediary), aspartic acid (an amino acid synthesised from oxaloacetate and a citric acid cycle intermediary) and uridine (a pyrimidine analog). ADGRL4/ELTD1′s suppression of the above metabolites suggests a suppressive effect on glycolysis and metabolites related to the TCA cycle. Interestingly, ACLY expression positively regulates glycolysis (with ACLY silencing leading to a decrease in glycolysis) [19], as does fructose 2,6 diphosphate [25]. ADGRL4/ELTD1′s repressive effect on both fructose 2,6 diphosphate and ACLY suggests a possible role for ADGRL4/ELTD1 in regulating glycolysis.

In the LC-MS experiments, the majority of metabolites were suppressed with the most suppressed being phosphocreatine (a high energy phosphate metabolite), N-acetylaspartyglutamate (a metabolite derived from glutamate), deoxyadenosine triphosphate (a nucleotide involved in DNA synthesis), and sedoheptulose 1-phosphate (a pentose phosphate pathway intermediary). Pathway analysis showed significant enrichment of metabolites associated with pyrimidine metabolism, amino acid metabolism pathways (alanine, aspartate, and glutamine metabolism; cysteine and methionine metabolism; taurine metabolism; and arginine and proline metabolism) and amino and nucleotide sugar metabolism. The metabolomics experiments were limited by the low number of significant FDR-adjusted *p* values, despite substantial FC effects. This is most likely related to the relatively low number of biological replicates (five). Future studies will use larger numbers of replicates to investigate whether metabolite changes are dependent on the expression of induced/repressed transcripts downstream of ADGRL4/ELTD1 expression.

ADGRL4/ELTD1 silencing upregulating KIT mRNA and surface protein is interesting as KIT has an important role in haematopoiesis and endothelial development. In haematopoiesis, KIT is expressed by early haematopoietic progenitor cells (HPC) (regulating haematopoietic stem cell (HSC) renewal as well lineage differentiation [26]) and within the haemogenic endothelial niche by haemogenic endothelial cells (HECs) [27]. Interestingly, ADGRL4/ELTD1 is part of a larger transcriptional programme regulating the differentiation of HECs to HSCs [28]. Together these results suggest new ways in which ADGRL4/ELTD1 regulates the endothelial phenotype.

Our findings reveal new relationships between ADGRL4/ELTD1 and the Notch pathway (*JAG1* and *HES2*). The findings regarding DLL4 confirm the previously documented relationship between ADGRL4/ELTD1 and DLL4 [1]. DLL4-Notch (which is repressed by ADGRL4/ELTD1 [1]) decreases endothelial glycolysis and mitochondrial respiration and increases endothelial exogenous fatty acid uptake [29]. By contrast, VEGFA (which induces ADGRL4/ELTD1 [1]) increases glycolysis [20]. ADGRL4/ELTD1′s hypothesised regulatory effect on glycolysis is thus likely to be nuanced and complex. KIT signalling is known to induce fatty acid metabolism through the transcriptional coactivator PPARGC1A, which upregulates fatty acid synthesis pathway genes [30].

In summary, this work establishes ADGRL4/ELTD1 as a regulator of endothelial metabolism through its suppression of *ACLY* and *SLC25A1* and its effect on various metabolites. It further extends ADGRL4/ELTD1′s relationship with the Notch signalling pathway and shows a new relationship with KIT. New insights into ADGRL4/ELTD1′s function within endothelial biology, and new experimental targets within the endothelial tumour microenvironment are clinically relevant findings that could be investigated as a means to better treat cancer.

## 4. Materials and Methods

### 4.1. Cell Lines and Maintenance

All cells were cultured in tissue culture incubators at 37 °C with a humidified atmosphere of 5% CO_2_ and 95% filtered atmospheric air. HUVECS pooled from multiple donors (Lonza, Wokingham, UK) were cultured in EBM-2 (Lonza) supplemented with EGM-2 (Lonza). HEK293T cells (Thermo Scientific, Leicestershire, UK) were cultured in DMEM supplemented with 10% FCS and 1% l-glutamine (Lonza). The medium was changed every 48 h for all cells.

### 4.2. Western Blotting

Cell lysate protein concentrations were quantified and resolved using SDS-PAGE (Invitrogen, Paisley, UK) and transferred to a PVDF membrane (Millipore, Livingstone, UK). Membranes were blocked for 1 h with 5% non-fat dry milk in 150 mM NaCl, 10 mM Tris, and 0.1% Tween-20 pH 7.5 (TBS-T). Primary antibodies were added overnight at 4 °C. Primary antibodies used were ELTD1 (Sigma HPA025229) 1:1000; ACLY (Abcam ab40793) 1:1000 (Abcam, Cambridge, UK); SLC25A1 (Abcam ab174924) 1:1000 (Abcam); β-actin conjugated to HRP (Sigma 3854) 1:10000 (Sigma, Welwyn Garden City, UK); and acetylated-lysine (Cell Signaling #9441) 1:1000 (Cell Signaling, London, UK). After washing a secondary antibody was applied, which was anti-rabbit conjugated to HRP (DAKO P044801-2) 1:2500 (DAKO, Cambridge, UK). ECL Prime (GE Healthcare, Chalfont St Giles, UK) was used to detect membrane immunoreactivity. Visualisation was performed using an ImageQuant LAS4000 (GE Healthcare).

### 4.3. RNA Analysis and qPCR

RNA extraction was performed using an RNeasy Mini Kit (Qiagen, West Sussex, UK). A High Capacity cDNA Reverse Transcription kit (Applied Biosystems, Cheshire, UK) was used for reverse transcription. qPCR was performed using a SensiFAST SYBR No-ROX kit (Bioline, London, UK). All kits were used according to manufacturer guidelines. qPCR primers were designed using Primer-BLAST [31] and were synthesised by Invitrogen. qPCR was run using the 7900HT Fast Real-Time PCR System (Applied Biosystems) in a 384-well format. Primers are listed in Supplementary Methods Table S1.

### 4.4. Flow Cytometry

Cells were prepared for FACS using standardised FACS methods. Primary antibodies were applied to cells for 30 min in a buffer comprising PBS and 3% FBS followed by washing and application of a secondary antibody for 30 min, followed by washing. Measurement was performed using a BD FACSCalibur flow cytometer (BD Biosciences, Wokingham, UK). Primary antibodies are listed in Supplementary Methods Table S2. Data was analysed using FlowJo 10.0.7 (FlowJo LLC, Ashland, USA).

### 4.5. RNA Interference

Transfection of siRNA duplexes was performed using Lipofectamine RNAiMAX (Invitrogen, MA, USA) according to the manufacturer’s instructions. siRNAs used were ADGRL4/ELTD1 siRNA 1 (5′-GGGCUGUGAGCUGACAUACUCAAAU-3′) (Invitrogen HSS148831) and ADGRL4/ELTD1 siRNA 2 (5′- CAAUCAUUGCCGGACUGCUACACUA -3′) (Invitrogen HSS184653). A medium GC negative control siRNA was used as ADGRL4/ELTD1′s siRNAs were all medium GC in content (Invitrogen 12935300). siRNAs were used at a final concentration of 20 nM. HUVECs were cultured in antibiotic-free medium prior to transfection and were assayed for ADGRL4/ELTD1 silencing (qPCR, Western blotting, and FACS) at 24 or 48 h post transfection.

### 4.6. Overexpresssion

ADGRL4/ELTD1 overexpression was performed by transducing low passage HUVECs with an ADGRL4/ELTD1 containing lentivirus using the Mammalian Expression System with Gateway Technology (Invitrogen) as per the manufacturer’s protocol. An empty lentivirus was used as the control. Cells were assayed for overexpression using qPCR.

### 4.7. Lipid Droplet Assays

Lipid droplets were stained with LipidTOX Green neutral lipid stain (Invitrogen H34475). For lipid droplet confocal microscopy, HUVECS were plated within confocal-appropriate 24-well plates with opaque walls (Greiner Bio-One International, Stonehouse, UK) followed by ADGRL4/ELTD1 silencing for 48 h. At 24 h, oleic acid in bovine albumin (Sigma Aldrich: O3008) was added to the positive control well (final concentration 150 μM). At 48 h, cells were exposed to DAPI (300 nM) (Invitrogen) for 10 min. Cells were then exposed to LipidTOX Green (1:100) for 30 m and imaged using a Zeiss LSM 780 confocal microscope (Zeiss, Cambridge, UK) (Alexa Fluor 488 filter-set was used). A 60× oil lens was used with laser strength adjusted based on results from negative/positive controls. Every well imaged had a minimum of six images taken. Images were analysed using FIJI [32]. For lipid droplet FACS analysis, HUVECs were silenced as above followed by harvesting and exposure to LipidTOX Green (1:100) for 30 m in the dark on ice. Cells were imaged live using FACS (capturing fluorescence in the Alexa Fluor 488 range).

### 4.8. Acetylation

HUVECs underwent ADGRL4/ELTD1 silencing as described. At 24 h, Panobinostat (Cayman Chemical: 13280), a pan HDAC inhibitor, was added (final concentration 25 nM). DMSO was used as a control. Cells were harvested at 48 h and probed for acetylation using Western blotting (antibody: anti-acetylated-lysine (Cell Signaling 9441)).

### 4.9. Metabolomics

Five unique batches of pooled HUVECs were plated into 10 cm dishes (7.5 × 10^5^/dish) and cultured under normoxic conditions for 24 h. ADGRL4/ELTD1 silencing was then performed as described above. Cells were harvested at 48 h and prepared for LC-MS using a standardised protocol (available on request). Each sample was analysed using three separate LC-MS methods using two different LC systems (Thermo Scientific ICS-5000 plus ion chromatography system and a Thermo Ultimate 3000). Each was coupled directly to a Q-Exactive HF Hybrid Quadrupole-Orbitrap mass spectrometer with a HESI II electrospray ionisation source (Thermo Scientific, San Jose, CA, USA).

Ion-exchange chromatography mass spectrometry was performed using a ICS-5000 plus HPLC system incorporating an electrolytic anion generator (KOH) which was programmed to produce a OH^–^ gradient over 37 min (this was based on the method published by Riffelmacher et al. [33]). An inline electrolytic suppressor removed OH^–^ ions and cations from the post-column eluent stream prior to MS analysis (Thermo Scientific Dionex AERS 500). A 10 μL partial loop injection was used for all analyses and the chromatographic separation was performed using a Thermo Scientific Dionex IonPac AS11-HC 2 × 250 mm with a 4 μm particle size column and a Dionex Ionpac AG11-HC 4 μm 2 × 50 guard column inline. The IC flow rate was 0.250 mL/min. The total run time was 37 min and the hydroxide ion gradient was comprised as follows: 0 min, 0 mM; 1 min, 0 mM; 15 min, 60 mM; 25 min, 100 mM; 30 min, 100 mM; 30.1 min, 0 mM; and 37 min, 0 mM. Analysis was performed in negative ion mode using a scan-range from *m/z* 60–900 and resolution set to 70,000. The tune file source parameters were set as follows: sheath gas flow 60 mL/min; aux gas flow 20 mL/min; spray voltage 3.6 V; capillary temperature 320 °C; S-lens RF value 70; and heater temperature 350 °C. The AGC target was set to 1e6v ions and the maximum IT value was 250 ms. The column temperature was kept at 30 °C throughout the experiment. Full scan data were acquired in continuum mode.

C18 reversed-phase analysis of underivatised samples was performed using a Thermo Utimate 3000 UHPLC system with a gradient elution program coupled directly to a Q-Exactive HF Hybrid Quadrupole-Orbitrap mass spectrometer running in negative ion mode. A 5 μL partial loop injection was used for all analyses with a pre- and post-injection wash program. A Waters HSS T3 1.6 µm (2.1 × 100 mm) column was used with a flow rate of 0.4mL/min. The total run time was 18 min. Mobile phase A comprised milli-Q water with 0.1% formic acid and mobile phase B was 100% methanol with 0.1% formic acid. The gradient elution program was as follows: 0 min, 5% B; 4 min, 50% B; 12 min, 99% B; 15 min, 99% B; 15.1 min, 5% B; and 18 min, 5% B. The column temperature was kept at 40 °C throughout the experiment. Mass spectrometry analysis was performed in positive and negative ion modes separately using a scan-range from *m/z* 60–900 and resolution set to 70,000. The tune file source parameters were set as follows: sheath gas flow 60 mL/min; aux gas flow 20 mL/min; spray voltage 3.6 V; capillary temperature 320 °C; S-lens RF value 70; heater temperature 350 °C. The full MS settings were AGC target 5e6 ions and the maximum IT value was 120 ms. Full scan data were acquired in continuum mode. A data-directed tandem mass spectrometry method (ddMS^2^) was utilised with no inclusion list. The orbitrap detector and HCD setting for ddMS^2^ were as follows: microscans 2, resolution 17,500, AGC target 5e4 ions, maximum IT 80 ms, loop count 10. and NCE 35.

The third LC-MS method used a sample derivatisation protocol followed by analysis based on a modified version of the Waters AccQ-Tag method [34]. C18 reversed-phase analysis of derivatised samples was also performed using a Thermo Utimate 3000 UHPLC system coupled directly to a Q-Exactive HF Hybrid Quadrupole-Orbitrap mass spectrometer. A 5 μL partial loop injection was used for all analyses with a pre- and post-injection wash program. A Waters AccQ-Tag column (2.1 × 100 mm) was used with a flow rate of 0.5 mL/min. The total run time was 9.5 min. Mobile phase A and B comprised commercially available AccQ-Tag reagents prepared as recommended by Waters (Waters PLC, Elstree, UK). The gradient elution program was modified from the published AccQ-Tag method as follows: 0 min, 0.1% B; 0.54 min, 9.1% B; 5.74 min, 21.2% B; 7.74 min, 59.6% B; 8.04 min, 90% B; 8.05 min, 90% B; 8.64 min, 0% B; and 9.5 min, 0.1% B. The column temperature was kept at 40 °C throughout the experiment. Mass spectrometry analysis was performed in positive ion mode separately using a scan-range from *m/z* 70–1050 and resolution set to 70,000. The tune file source parameters were set as follows: sheath gas flow 60 mL/min; aux gas flow 20 mL/min; spray voltage 3.6 V; capillary temperature 320 °C; S-lens RF value 70; and heater temperature 350 °C. The full MS settings used were AGC target 3e6 ions and the maximum IT value was 200 ms. Full scan data were acquired in continuum mode.

Data analysis was performed using Progenesis QI (Non-Linear Dynamics, Elstree, UK). Thresholds were set at *p* < 0.05 and FC > 1.2. Pathway analysis was performed using MetaboAnalyst. Over-representation analysis and pathway topology analysis was performed on significantly modified metabolites in MetaboAnalyst using hypergeometric testing and relative-betweenness centrality testing, respectively. [35]. Data was normalised by sum and Pareto scaled.

### 4.10. Microarray Analysis

Gene expression profiling analysis of ADGRL4/ELTD1-silenced HUVECs was performed by the Wellcome Trust Centre for Human Genetics (Oxford) using a HumanHT-12 v4 Expression BeadChip Kit (Illumina, Cambridge, UK) and Illumina microarray scanner (Illumina iScan). Raw data was pre-processed using R (version 3.3.2) and Limma (Smyth, 2004). Differentially expressed genes were deemed statistically significant if they fulfilled the standard microarray criteria: (i) an adjusted *p* value < 0.05 and (ii) a fold change of > 2 < −2. Pathway analysis was performed using “Enrichr” [36] “KEGG” [37], “Reactome” [38], “Wikipathways” [39], and “BioCarta” [40]. The false discovery rate threshold was set to 0.05. Visualisation was performed using “GGplot2” [41] and Morpheus. All microarray experiments were MIAME compliant [42].

### 4.11. Statistical Analyses Using Prism

Results from all completed wet-lab experiments were analysed using Prism 7 (Graphpad Software). For comparison of two unpaired groups, an unpaired Student’s *t*-test was used. Significance was denoted as * *p* ≤ 0.05, ** *p* ≤ 0.01, *** *p* ≤ 0.001, and **** *p* ≤ 0.0001.

## Supplementary Materials

The following are available online at https://www.mdpi.com/2218-1989/9/12/287/s1, Figure S1: Validation of additional microarray targets, Figure S2: HUVEC ADGRL4/ELTD1 silencing does not affect fatty acid synthesis pathway genes, Figure S3: Maximum fold change for selected metabolites (*p* ≤ 0.05 and fold change > 1.2), Table S1: Metabolite pathway analysis with extended range of metabolites (FC > 1.2 irrespective of *p* value), Supplementary Methods Table S1: qPCR primer sequences, and Supplementary Methods Table S2: FACS antibodies.

## Abbreviations

7TM	7-transmembrane domain
AAA	α-Aminoadipic acid
aGPCR	adhesion GPCR
CTF	C terminal fragment
CT	control
EMT	endothelial to mesenchymal transition
EPC	endothelial progenitor cell
FACS	fluorescence-activated cell sorting
GAIN	GPCR autoproteolysis inducing domain
GPCR	G protein-coupled receptor
GPS	GPCR proteolysis site
HPC	haematopoietic progenitor cell
ICD	intracellular domain
LC-MS	liquid chromatography-mass spectrometry
NTF	N terminal fragment
TCA	trichloroacetic acid/acetone
WT	wild type
