# Supplementary material for: ADGRL4/ELTD1 Silencing in Endothelial Cells Induces ACLY and SLC25A1 and Alters the Cellular Metabolic Profile
Source: Metabolites. 2019 Nov 25;9(12):287. doi: 10.3390/metabo9120287 (PMC6950702; doi:10.3390/metabo9120287)
Supplement: Supplementary file 1 [file metabolites-09-00287-s001.zip › Supplementary Materials.pdf]

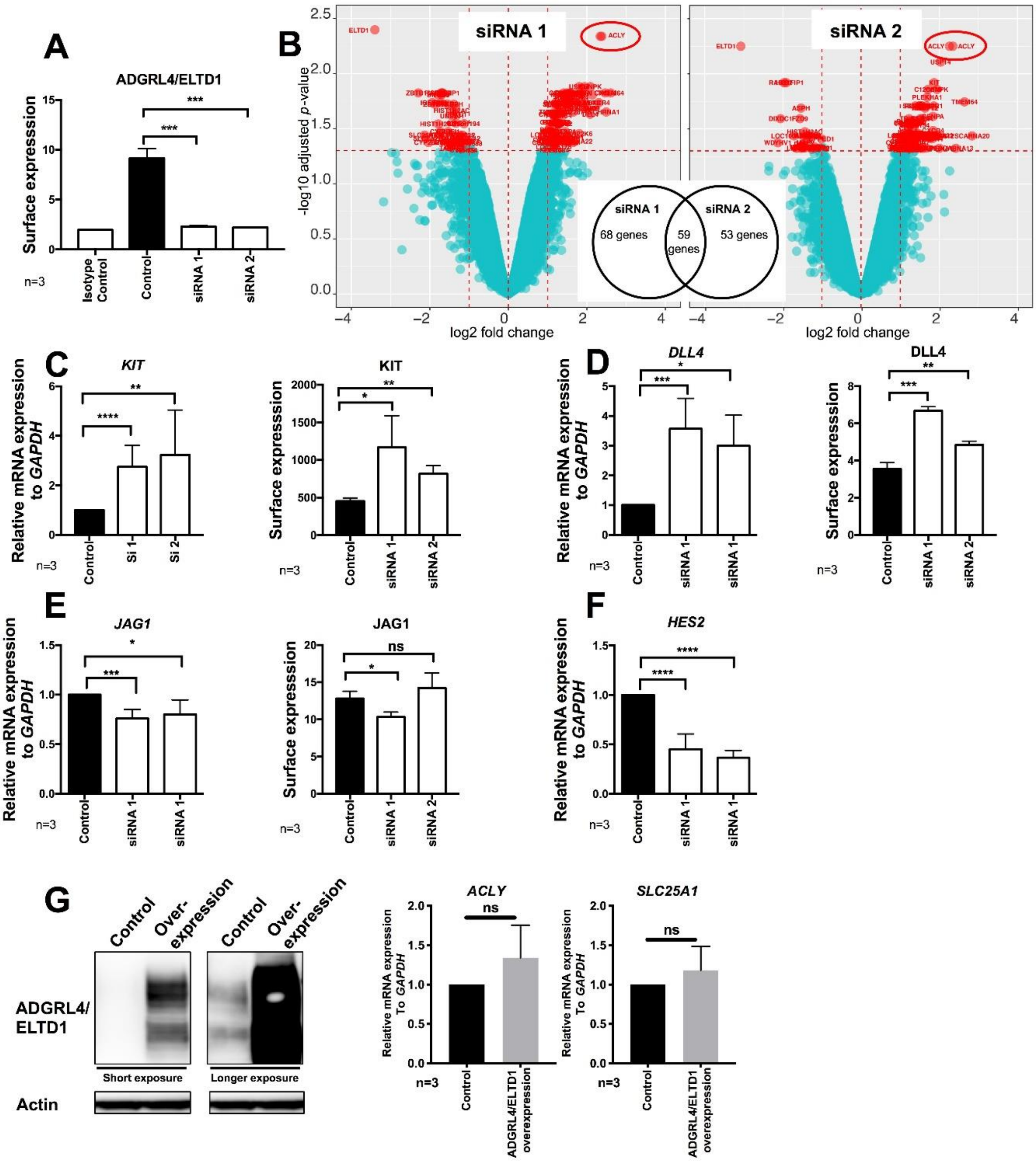

Figure S1. Validation of additional microarray targets (A) Quantification of FACS ADGRL4/ELTD1 surface. **(B)** Volcano plots and Venn diagram depicting differentially expressed genes for each siRNA.**(C)** *KIT* qPCR and FACS quantification of surface KIT protein expression. **(D)** *DLL4* qPCR and FACS quantification of surface DLL4 protein expression. **(E)** *JAG1* qPCR and FACS quantification of surface JAG1 protein expression. (**F)** *HES2* qPCR. **(G)** Representative western blot showing ADGRL4/ELTD1 overexpression in HUVECs (longer exposure, on the right, required to show endogenous expression in wild type HUVECs) and *ACLY* and *SLC25A1* qPCR quantification in ADGR4/ELTD1 overexpressing HUVECs. (* $p \leq 0.05$, ** $p \leq 0.01$, *** $p \leq 0.001$, **** $p \leq 0.0001$)

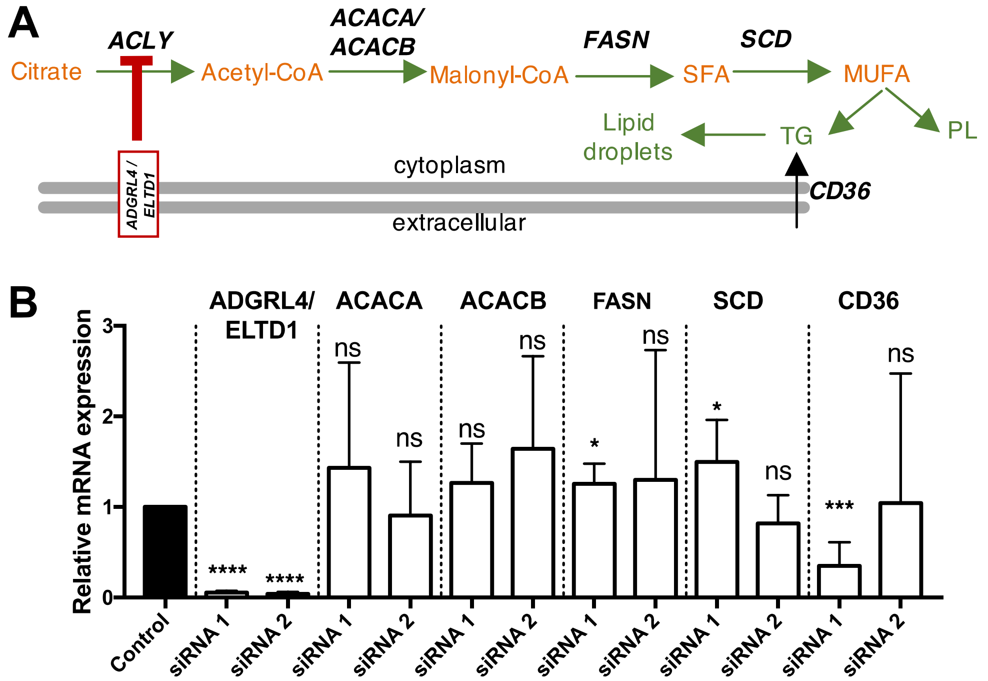

Figure S2. HUVEC ADGRL4/ELTD1 silencing does not affect fatty acid synthesis pathway genes. **(A)** The fatty acid synthesis and cholesterol pathways. Key fatty acid synthesis enzymes are in black and substrates are in orange. ADGRL4/ELTD1 labelled in red. **(B)** Fatty acid synthesis pathway qPCR results. (* $p \leq 0.05$, ** $p \leq 0.01$, *** $p \leq 0.001$, **** $p \leq 0.0001$) (Abbreviations: MUFA= monounsaturated fatty acid; PL=phospholipid; SFA=saturated fatty acid; TG=triglyceride).

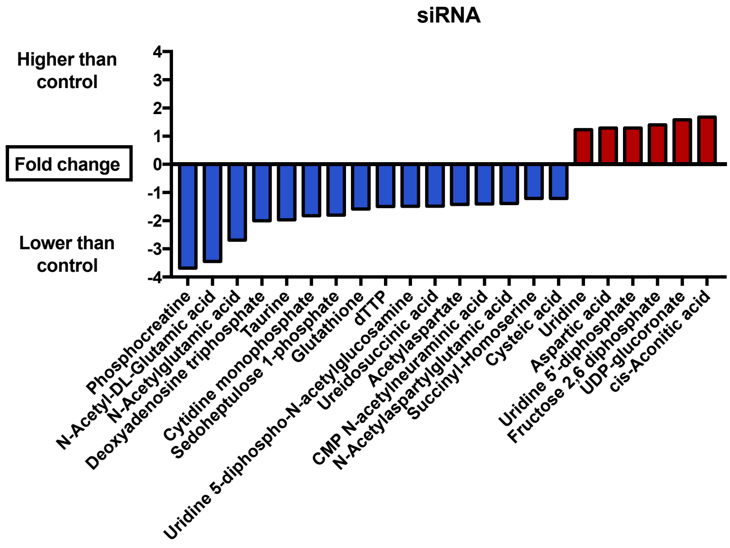

Figure S3: Maximum fold change for selected metabolites ($p \leq 0.05$ and fold change > 1.2).

Pathway Name	Match	*p*	FDR	Metabolites
Alanine, aspartate and glutamate metabolism	6/24	0.000002	1.67E-04	N-Acetyl-L-aspartic acid, L-Aspartic acid, Oxoglutaric acid, L-Glutamine; Gamma-Aminobutyric acid; Fumaric acid;
Pyrimidine metabolism	8/60	0.000005	2.25E-04	Uridine 5'-diphosphate; dCTP; L-Glutamine; Uridine; Cytidine monophosphate; Thymidine 5'-triphosphate; Thymidine; Malonic acid;
Arginine and proline metabolism	7/77	0.00004	0.007612	L-Glutamine; L-Aspartic acid; L-Arginine; N-Acetyl-L-alanine; Phosphocreatine; Gamma-Aminobutyric acid; Fumaric acid
beta-Alanine metabolism	4/28	0.001	0.024222	Malonic acid; L-Aspartic acid; Gamma-Aminobutyric acid; L-Histidine
Nitrogen metabolism	4/39	0.004	0.061099	Taurine; L-Aspartic acid; L-Glutamine; L-Histidine;
Citrate cycle (TCA cycle)	3/20	0.004	0.061099	Oxoglutaric acid; cis-Aconitic acid; Fumaric acid;
D-Glutamine and D-glutamate metabolism	2/11	0.01	0.15157	L-Glutamine; Oxoglutaric acid
Cysteine and methionine metabolism	4/56	0.01	0.15157	O-Succinyl-L-homoserine; Glutathione; Cysteic acid; L-Aspartic acid
Amino sugar and nucleotide sugar metabolism	5/88	0.01	0.15157	Uridine diphosphate glucuronic acid; Uridine diphosphate-N-acetylglucosamine; Glucose 1-phosphate; Cytidine monophosphate N-acetylneuraminic acid; Uridine diphosphategalactose;
Butanoate metabolism	3/40	0.03	0.24117	Gamma-Aminobutyric acid; Oxoglutaric acid; Fumaric acid
Galactose metabolism	3/41	0.03	0.24117	Glucose 1-phosphate; Uridine diphosphategalactose; Sorbitol
Aminoacyl-tRNA biosynthesis	4/75	0.03	0.24117	L-Histidine; L-Arginine; Glutamine; L-Aspartic acid;
Histidine metabolism	3/44	0.04	0.24117	L-Histidine; L-Aspartic acid; Oxoglutaric acid;
Ascorbate and aldarate metabolism	3/45	0.04	0.24117	Uridine diphosphate glucuronic acid; D-Glucarate; Oxoglutaric acid;
Taurine and hypotaurine metabolism	2/20	0.04	0.24699	taurine; cysteic acid
Pentose and glucuronate interconversions	3/53	0.06	0.31668	D-Xylulose; Glucose 1-phosphate; Uridine diphosphate glucuronic acid
Glycolysis or Gluconeogenesis	2/31	0.1	0.47174	L-Aspartic acid; Fumaric acid

Table S1: Metabolite pathway analysis with extended range of metabolites (FC >1.2 irrespective of *p* value).

Supplementary dataset

supplementary_raw_output_metabolomics.xlsx

Supplementary methods

Gene name	Forward primer (5′-3′)	Reverse primer (5′-3′)
ACACA	AACGTGAAGACGGATAAGCAG	CTTCTTCGGAGAATCTGACCA
ACACB	AGTTCGCCGACTTCCAT	TCTTCCACTCCAGGATGTCA
ACLY	CCAGCAGGACAGCATCTTTT	GGATCTTGGACTTGGGACTG
ACTB	ATGCTCTCCCTCACGCCATC	CACGCACGATTTCCCTCTCA
CD36	GTGCCTATTCTTTGGCTTAATGA	TTACTTGACTTCTGAACATGTTTGC
DLL4	CCCTGGCAATGTACTTGTGAT	TGGTGGGTGCAGTAGTTGAG
ADGRL4/ELTD1	GCTCAAACCCACCCACATTAT	CACAGCCCTCTGAAGACCAG
FASN	CAGGCACACACAGGATGGAC	CGGAGTGAATCTGGGTTGA
GAPDH	AGCCACATCGCTCAGACAC	GCCCAATACGACCAAATCC
HES2	CCAACTGCTCGAAGCTAGAGA	AGCGCACGGTCATTTCCAG
JAG1	GAATGGCAACAAAACTTGCAT	AGCCTTGTCGGCAAATAGC
KIT	AATCCTCTCGTCAAAACTGAAGG	CCATCTCGCTTATCCAACAATGA
SCD	GACCTGCTTGCTGATCCTGT	CCATTCACGAAGCATCTCAC

Supplementary methods Table 1: qPCR primer sequences.

Target	Type	Dilution	Company

**Primary antibodies**			
ADGRL4/ELTD1	Mouse IgG2 monoclonal	10 μg/mL	Professor Alison Banham, Oxford University (184.11)
DLL4	Rat IgG2 monoclonal	10 μg/mL	R&D Systems (MAB1506)
JAG1	Mouse IgG2 monoclonal	1 in 200	Professor Alison Banham, Oxford University (J1-65D)
KIT	Mouse IgG1 monoclonal	1 in 200	Invitrogen (17-1178-41)
IgG1 isotype control	Mouse IgG1 monoclonal	10 μg/mL	Invitrogen (17-4714-41)
IgG2 isotype control	Mouse IgG2 monoclonal	10 μg/mL	Invitrogen (17-4724-41)
**Secondary antibodies**			
anti-mouse APC	Goat IgG	1 in 100	Invitrogen (A-865)
anti-rat APC	Goat IgG	1 in 100	Invitrogen (A10540)

Supplementary methods Table 2: FACS antibodies.
